# The connected patient project: moving towards a population-based primary health care research registry

**DOI:** 10.1186/s12913-021-06486-1

**Published:** 2021-05-31

**Authors:** Dorothy Coe, Angela Birt, Gareth Forbes, Jonathan Ling, Michael Foster, Stephen Robson, Joe McDonald, Yan Yiannakou

**Affiliations:** 1 National Institute for Health Research, Clinical Research Network North East North Cumbria, Newcastle Upon Tyne, UK; 2 Leadgate Medical Practice, Consett, UK; 3 University of Sunderland, Sunderland, UK; 4 University of Newcastle, Newcastle Upon Tyne, UK; 5 Connected Health Cities, Newcastle Upon Tyne, UK; 6 University of Teesside, Middlesbrough, UK; 7 University Hospital North Durham, North Road, Durham, DH1 5TW England

**Keywords:** Patient registries, Research recruitment, Digital communication, Data-sharing

## Abstract

**Background:**

The NHS pledges to give all patients access to clinical research. In England, 32% of General Practices are research active and only 14% of patients engage in research. This project aimed to evaluate consent-for-contact and communication in primary care patients.

**Methods:**

An explanatory mixed methods study of patients and staff within a single general practice. The study included all patients over the age of 18 years, and excluded those on the palliative care register and those unable to give informed consent. The questionnaire asked recipients to indicate their preferred contact method and data-sharing permissions with three organisations: NHS, Universities and Commercial Companies. Survey recipients and staff were invited to take part in a semi-structured interview. Interviews explored project acceptability, feasibility and reasoning behind choices made. Statistical data were triangulated with interview data.

**Results:**

The target patient population was 4678, 24% (*n* = 1148) responded. Seven hundred and three gave permission for at least one of the organisations to contact them. Older people were more likely to respond than young people, (*p* < 0.001). There was a trend for more women than men to give permissions however, in the 70 years plus age group this was reversed. Short message service was the preferred method of communication (48% *n* = 330), but those aged 70 years and over, preferred letter (*p* = 0.001). Interviews suggested patients felt the project was primarily about improving communication and secondly access to research. Patients trusted the NHS and university researchers. Staff interviewees found the project was less onerous than expected. Barriers to wider rollout included workload and the fragmented nature of NHS digital systems.

**Conclusions:**

A registry of patients was established; however, the response rate of 24% needs increasing before wider adoption. Health promotion and chronic disease-based research may recruit better when based in primary health care. Older demographics would be more likely to volunteer for research. NHS and academic researchers are trusted, commercial organisations less so. The move to digitalise communication methods has the potential to marginalise older women. Findings were used to drive forward two novel developments: a consent registry (Research+Me) and a federation-wide participant identification process.

## Background

The NHS pledges to allow every patient access to research [1]. There are known benefits to engaging in research, despite this only 14% of patients participate, and only 32% of General Practices in England are currently research active [2, 3]. To address this, research capacity and capability is being investigated and developed in primary health care (PHC). Studies of chronic conditions commonly recruit participants from General Practice, often using the site as a Participant Identification Centre (PIC) [4]. This process has multiple steps undertaken by individual practices by staff for whom research is not their primary role or objective. This can lead to delays in participant identification, inappropriate identification and reduce the numbers of potential participants [5]. Once participants are identified, the individual practice then sends each potential participant study information, normally by post. The PIC process has limitations which can lead to reduced opportunities for PHC patients to access research. It also leads to small numbers of interested practices being repeatedly contacted, which can create inequalities of access. This project offers an opportunity to explore other potential strategies to develop and improve the PIC process. As part of this, novel recruitment strategies are being explored, and different communications mediums suggested. The data-sharing and communication preferences of potential participants could be utilised to reduce the numbers of processes in the PIC system, speed up communications and widen the scope to increase the number of possible participants recruited from PHC.

There are known reservations from the public regarding data sharing [6–8]. Headlines about data breaches, use of personal data and cyber-attacks highlight the need for the public to be fully informed about why and how their identifiable information is used [8, 9] and confident that it will not be misused. Mistrust coalesces around issues regarding confidentiality, abuse of information and the competence of organisations to keep it safe. This project set out to test a method of constructing a primary health care-based patient registry. The overall aim was to increase the understanding of data sharing and communication preferences of patients in primary care, with a view to improve PIC processes and registry development.

This article will explore the viability of the method, discuss the profile of the local registry of patients and suggest additional areas that may aid research recruitment from PHC. To enable this, it will report quantitative and qualitative findings and the subsequent discussion following the synthesis of these data.

## Methods

### The connected patient project

The project was set in a General Practice in the North East of England. All eligible participants were sent a letter of introduction, information on data sharing, a postal survey questionnaire and a pre-paid reply envelope. The information covered data-sharing to improve medical care, the sharing of de-personalised information and the requirement to occasionally share information compulsorily. The survey focussed on the potential for sharing information directly with researchers to enable them to make direct contact regarding research projects of interest. An example was given which illustrated a university researcher, contacting the General Practice about research, involving people with a visual impairment aged 65 years or over. The example described the General Practice doing a computer search for those who had a visual impairment, 65 years or over and who had given permission for researchers from a university to contact them directly.

Participants were asked to indicate their permission preferences for the General Practice to share their personal contact details and relevant medical information with researchers from three different types of organisation (NHS, Universities, Commercial Companies). The information stated that this did not mean they had agreed take part in research, only that they would receive information about research before they decided to take part or not. To explore the acceptability of digital communication streams the recipients were asked to update their preferred mode of contact, the choices being, email, text (Short Message Service: SMS) or letter. They were requested to indicate their choices on the enclosed survey questionnaire and return it in the pre-paid envelope or access their personal online General Practice account and complete an electronic version. The participants were informed they did not have to fill out the survey if they did not want to, and that a researcher based in the practice was looking at the choices made to see if there were any patterns. If they did not want the researcher to use the anonymous information there was a section on the questionnaire to indicate this. Additionally, the information contained a section inviting participants to an interview to discuss their thoughts on the project and the sharing of information.

### Design

A sequential explanatory mixed methods [10] approach was taken. This allowed for two phases, an initial quantitative phase, followed by a qualitative phase to aid the explanation of the quantitative findings. The initial quantitative preference setting stage was conducted via a postal survey questionnaire. Returning the survey was taken as implied consent. The subsequent qualitative phase used interviews with both patients and staff. The interviews used topics guides to explore the findings from the survey and the processes undertaken.

The researcher based in the general practice evaluated the process of setting up a primary care-based registry by analysing statistical data from the practice clinical system, survey responses and interview data from patients and staff.

### Patient and public involvement

Prior to ethical review all participant-facing documentation was reviewed and approved by the patient reference group of the general practice. A summary of the findings has been posted on the general practice’s website.

### Sample

#### Inclusion criteria

All patients registered with the practice aged 18 years or over at the time of contact. All staff working within the practice (interviews).

#### Exclusion criteria

Patients under 18 years at the time of contact. Patients currently on the palliative care register. Patients unable to give an informed consent (interviews).

Total eligible sample patients was 4678, and the total eligible sample staff was 15.

### Data collection

#### Postal survey

Eligible patients received one copy of the survey pack. The cohort list was sorted by NHS number. Batches of 350–750 were sent out via a secure system across a 14-week period. The survey could be returned in person, via a pre-paid envelope or via the individual’s practice based online account. Quick Response (QR) codes were added which linked to the online account login page and additional information. Advice was given on how to set-up an online account. The survey contained statements to which the recipient indicated their chosen response. The contact preference statement was: ‘*my preferred way for the practice to contact me about non-urgent things is’;* there were then 3 choices, text, email, letter, with a tick box against each choice. The statement regarding contact was phrased *‘I give you permission to share my personal information with NHS researchers so they can contact me about research’.* Each statement was then repeated with a change of organisation to university researchers or commercial researchers. After each statement there was a yes or no tick box.

Data were entered into Microsoft Excel and added to the clinical system to allow cross referencing of survey responses and demographic characteristics. Permissions and contact choices were added to the individual’s record. There were eight possible permission responses (see Table 1). Baseline data collected from the clinical system included, the number of verified email addresses and mobile phone numbers held, plus the number of personal online accounts.

**Table 1:** possible combinations of permission responses

	NHS Researchers	University Researchers	Commercial Researchers	Acronym
Possible responses	Yes	Yes	Yes	YYY
	Yes	Yes	No	YYN
	Yes	No	No	YNN
	Yes	No	Yes	YNY
	No	Yes	Yes	NYY
	No	Yes	No	NYN
	No	No	Yes	NNY
	No	No	No	NNN

Qualitative data were collected from patients and staff via semi-structured interviews, using different topic guides for each group. Patient interviews used open questions to explore the reasoning behind the choices made, thoughts about data sharing and the practicality of the project design. Staff interviews examined the impact of the project with reference to the increase in workload, and the acceptability and feasibility of it being repeated in another setting. The survey material contained an open invite to all recipients to take part in an interview. In addition, ninety individual invites for interview were sent to a cross section of patient responders. These were purposefully selected [11] to reflect the age profile of the eligible cohort and range of responses given. All interviewees were volunteers and gave written informed consent. All interviews were conducted by the researcher working in the general practice at a time and place mutually convenient and were digitally recorded.

### Data analysis

#### Survey

Descriptive and inferential statistics in the form of chi-square tests were used to illustrate the findings. An alpha of *p* = 0.05 was set to determine statistical significance. Response rates were calculated and cross-referenced with the choices made, age group, sex and presence or absence of a long-term condition (LTC) as identified by the General Medical Services contract Quality and Outcomes Framework [12]. Digital connectivity was analysed by noting the response rate across the different routes and any changes from baseline in the number of mobile phone numbers, verified email addresses and personal online accounts.

#### Interview data

Interviews were transcribed verbatim and anonymised. The transcripts were thematically analysed using a six-step process [13]. This assisted in the production of a narrative to describe the findings and support inferences. The analysis began with familiarisation of the transcripts, followed by initial coding, extraction of statements of significant meaning and aggregation of themes. Each theme was supported by quotes from individuals [13, 14]. Themes were verified for trustworthiness by the core research team.

## Results

### Survey response rate

Of the total practice population of 5946 patients, 4678 were eligible and received the survey. Of those, 24% (*n* = 1148) responded to indicate their contact method and data sharing preferences.

Table 2 shows the age and sex profile of the responders.

**Table 2:** Demographic profile of responders

NHS	University	Commercial	Number of patients	18–29 yrs.	30–39 yrs.	40–49 yrs.	50–59 yrs.	60–69 yrs.	70+ yrs.	Men	Women
Yes	Yes	Yes	378	18 = 5% (5 m 13w)	25 = 7% (9 m 16w)	43 = 11% (23 m 20w)	71 = 19% (31 m 40w)	96 = 25% (34 m 62w)	125 = 33% (65 m 60w)	167 44%	211 56%
Yes	Yes	No	244	8 = 3% (4 m 4w)	23 = 9% (5 m 18w)	25 = 10% (10 m 15w)	50 = 20% (13 m 37w)	71 = 29% (32 m 39w)	67 = 27% (29 m 38w)	93 38%	151 62%
Yes	No	No	75	3 = 4% (0 m 3w)	6 = 8% (2 m 4w)	5 = 7% (3 m 2w)	18 = 24% (8 m 10w)	16 = 21% (9 m 7w)	27 = 36% (12 m 15w)	34 45%	41 55%
Yes	No	Yes	3						3 = 100% (3 m 0w)	3 100%	
No	Yes	Yes	0								
No	Yes	No	3			1 = 33% (1 m 0w)		1 = 33% (0 m 1w)	1 = 33% (1 m 0w)	2 67%	1 33%
No	No	Yes	0								
No	No	No	445	22 = 5% (12 m 10w)	35 = 8% (12 m 23w)	54 = 12% (26 m 28w)	79 = 18% (40 m 39w)	117 = 26% (55 m 62w)	138 = 31% (58 m 80w)	203 46%	242 54%
			**1148**	**51** **(4%)**	**89** **(8%)**	**128** **(11%)**	**218** **(19%)**	**301** **(26%)**	**361** **(34%)**	**502 (44%)**	**646 (56%)**

#### Age/sex profile

Response rate was higher in women (26%) than men (22%) (*p* = 0.001). Increasing age was also associated with higher response; of the eligible over 60 years age group 58%, (*n* = 662) responded, conversely 4% (*n* = 51) of the eligible 18–29 years age group responded. This age difference in response rate was statistically significant (*p* < 0.001).

### Permissions preferences

Of those who responded, 61% (*n* = 703) gave permission to be contacted by at least one of the three organisations, equating to 15% of the total eligible practice population. The most popular organisation was the NHS, with 61% (*n* = 700), followed by universities 54% (*n* = 625) with commercial at 33% (*n* = 381). The remaining 39% (*n* = 445) refused to give permission to contact from any organisation.

Of women responders, 62.5% (*n* = 404) said yes to one or more organisations. This rate was lower, 59.6% (*n* = 299) for men responders, but the difference was not statistically significant (*p* = 0.30). However, in the 70 plus age group this was reversed: men were more likely, 65.5% (*n* = 110) to say yes to one or more organisations than were women, 58.5% (*n* = 113). Again, this difference is not statistically significant (*p* = 0.18), but may indicate a trend that requires further investigation.

#### Permissions preferences and LTC

Of the eligible cohort 41% had a LTC. Of all responders 60% (*n* = 692) had a LTC and 61% (*n* = 231) of Yes, Yes, Yes (YYY) responders had a LTC. Having a long-term condition did not impact on the permissions choices made.

### Contact preferences

Contact method was related to age. Whilst text (SMS) messaging was the preferred overall mode of contact (48% *n* = 330), more subjects in the 70+ age group preferred letter contact (60% (*n* = 124), *p* < 0.001). Email was the least popular choice (20% *n* = 134). Data taken from the clinical system prior to, and post the survey, revealed there had been a 2% (3613 to 3716) increase in mobile phone numbers held and verified email accounts had increased from 15 prior to the survey to 185 post the survey.

### Patient interviews

Nine patients volunteered to be interviewed. All interviewees responded to the personal invite sent directly from the researcher. Average interview time was 26 min. A detailed description of the participants is given in Table 3. All had mobile phones; some were only used for emergencies. Eight of the interviewees had previous experience of research.

**Table 3:** Description of the nine patient interviewees

Participant	Age group	Sex	Contact choice	Permissions choices	Long-term condition	Online account	Social media use
**1**	50-59 yrs.	Women	Email	YYY	Yes	Yes/uses	Limited
**2**	50-59 yrs.	Man	Email	YYN	No	No	Limited
**3**	70 yrs. +	Women	Email	YYY	Yes	Yes/does not use	No
**4**	70 yrs. +	Women	Text	YYN	Yes	No	No
**5**	60-69 yrs.	Man	Letter	YNN	Yes	Yes/uses	Limited
**6**	40-49 yrs.	Women	Text	YYN	Yes	Yes/does not use	No
**7**	60-69 yrs.	Women	Email	YNN	No	Yes/does not use	No
**8**	60-69 yrs.	Man	Email	YNN	Yes	Yes/uses	No
**9**	60-69 yrs.	Women	Email	YNN	Yes	No	Limited

From the patient interview data four themes, which broadly follow the areas explored via the topic guides were identified:

#### Understanding

Five of the patients thought the project was more about communication between themselves, the practice and the wider NHS. ‘*I formed the impression that it was improving communication between the individual, the patient and the system’*, (Participant 3). Others suggested it was about research and gathering information and some had confused the different aspects.

*What I thought it was about extending research, so the form, there was a fair bit of detail about confidentiality and other things, but the gist I thought was about basic research and I took it to be expanding the ability to gather information, so that’s what I took it to be about.* (Participant 2).

W*ell, what I didn’t appreciate was that the means of communication was the purpose of the research I thought the project was something else, like putting my name forward for something else, something else like disease or drugs or something*. (Participant 1).

*I thought it was just general admin really, asking how they would, what my contact is, and then I wasn’t too sure about the researchers’ bit.* (Participant 8).

The majority felt the information could have been condensed and made clearer. The suggestion being that it may have been the volume of material that deterred some from responding.

*I thought it was quite wordy, I thought there was quite a lot of it and you had to sift through to find out what it was about.…. I still feel that the point of the exercise is lost in the procedural information. There you go* [turning the page] *it’s off putting, there are such a lot of words. If it was shorter you might have got more back*. (Participant 1).

Others stated that receiving the survey by post and directly from the practice engaged their interest and added to the bona-fide feel. ‘*It sort of makes you look at it, because it does looks so professional, it does look….it makes you engage with it, I’ve got to read this’*, (Participant 5).

Data sharing and research were regarded as necessary, being caveated with notions of confidentiality and anonymity. On the whole interviewees conflated the information given about the routine use of data in general practice with the information given about the permissions choices they were making and the sharing of personal contact details with researchers.

#### Motivations

All expressed altruistic motives and reciprocity. Their desire was to reciprocate by giving something back to the NHS, the practice and the wider society. This was linked to support and improvement of the NHS. An anti-privatisation rhetoric was expressed by some.

*Research to me, in my opinion or, how can I put it, could help another person with either one of my conditions, that’s why I said yes. They* [NHS] *have helped me stay alive all these years, and if I can help some other people… and if I can just give a little bit back with me trust not being abused*. (Participant 6).

*I’m very fond of my GP practice and anything I can do to assist them I feel is like a reasonable exchange for the services they offer me.* (Participant 1)

*I also feel quite strongly about the NHS, the way things are going and you know I would do anything to try and keep it, but I am very against the contracting out of things and also obviously the private, the push to privatise everything, I feel quite strongly about it.* (Participant 9).

Reciprocity, expressed by a desire to give something back to the wider society as well as the healthcare system, was associated with a health-related concern or cue to action. These were, LTC’s, aging and improved surveillance. There was a feeling that taking part in research would improve personal health outcomes.

*…you’ve got the age-related stuff you know, I’m a male over 50, so you know there are lifestyle things, general health things, so I thought it would be pretty all encompassing so I for all there might be some specific stuff there might be general as well. There is certain things in my family history if the knowledge had been better people might have been able to live longer or whatever, had certain treatments, it’s really down I guess partly to generic improvement and partly personal improvement, ‘cause there might be somebody contact me about something I’ve got that might help me in the long term that I know nothing ‘cos you don’t know what you’re talking about do you. Something might help you long term, but ultimately, it’s about just sharing information and I don’t see any harm in that.* (Participant 2).

*Well, I presume relevant to my age, my health situation, what am I doing controlling my blood pressure.* (Participant 3).

*Probably illnesses, research into lifestyle, so history, medical history, or those types of things*. (Participant 8).

Choice of contact method was motivated by convenience, previous use and the knowledge these were cheaper options for the NHS/GP surgery.

*I thought it was a good idea* [the project] *partly just the how you contact, I thought was quite good, to save money for the NHS…I hadn’t realised until recently how much a stamp is.* (Participant 8).

*I don’t use a mobile phone so text isn’t an option, and phone can be quite difficult sometimes, whereas I check email regularly.* (Participant 7).

#### Trust

Lack of trust is known to impact on the research process [15]. It is one of the major factors in the decision to participate in, and consent to research. For the participants in this project the NHS was a trusted brand; previous data breaches or cyber-attacks were not spoken about. It was suggested that the NHS operates up to date security systems and that data had not been lost or misused. Universities were also trusted but with less overt effusiveness. As both were perceived as not for profit, this increased their perceived trustworthiness. Trust and mistrust were thought of as a ‘gut reaction’. Trust was linked to anonymisation processes, confidentiality and the ability to change your mind and your personal permissions choices.

*Well, it* [the NHS]*, has integrity, public spirited, it’s for all people, whether rich or poor, yes it makes mistakes.* (Participant 3).

*In simple terms not for profit, in a nutshell, it’s not for profit, so you feel it’s been done for genuine reasons. It’s been done, someone is putting the effort in, Ok it’s got to be funded, but they’re not openly just out to make a couple of quid*. (Participant 2).

*Gut feeling, I think, it has to come down to, for me, despite the horrible documentary I watched on the TV last night about horror in some hospital, I trust the NHS, I think it is a wonderful thing really.* (Participant 1).

The word ‘commercial’ had a significant amount of mistrust attached to it. The phrases associated with commercial research were: ‘*money-making’, ‘selling data on’, ‘lack of impartiality’, ‘less honourable’, ‘promotional material’* and *‘not interested in people’.*

*I get loads of commercial contact and I just didn’t want to be bothered anymore and also, I think they would probably have their own axe to grind, they might not be as impartial as* [the] *NHS.* (Participant 8)

*I didn’t want to be caught up in a load of promotional stuff thereafter, their motives might be less honourable, involving money and finance and selling stuff to people. I just think that sometimes industry doesn’t do things for the best motive, and I fear that people’s personal details could be, not used against them, but used in a way that doesn’t suit me basically. (Participant 1)*

ProgressWhile participants were in favour of progress and a general movement towards SMS and digital contact, all felt letters should still be utilised. Use of smart phones was linked to difficulties in reading large amounts of text on a small screen. There was a hope that the ‘human face’ was not replaced by further digital interfaces.

*Not too digital please, the younger generation that is all they will know, but you have got to allow for the older people* [and] *gradually phase it* [in]. *(*Participant 7).

### Staff interviews

Five staff interviews were completed with the two members of staff who had worked on the project. To enable evaluative discussions across the phases of the project, staff involved were interviewed on more than one occasion. Four themes were identified.

#### Understanding of the project

The project was seen as a way of recognising the volume and diversity of the information PHC holds. Maximising the routes of communication and a way of ensuring the patients were informed and comfortable with how data was used.

*We want to make sure that patients are happy with how, they are happy with how we use their information, we want to maximise the ways that we communicate with them and them with us*. (Staff 2).

### Processes undertaken

Processes engaged with involved the updating of the clinical system, putting the survey onto the online portal and uploading the cohort details to a secure online postal service. Interviewees noted that the cohort list could be stratified by address to allow all members of the same household to receive the survey on the same day. This view was supported by the patient interviewees.

The functionality of clinical system SMS messaging was explored. Issues identified were, lack of nuance and richness, inability to insert hyperlinks and no information on which mobile phone numbers held would support *‘smartphone’* technology. Utilising email proved problematic. Issues included multi-person email addresses, currency of the email address, the lack of encryption and no specific policy or procedure to guide its use.

*Well, the difficulty with email is that you don’t know whether that account is active, you don’t know if the account exists in the first place, because I think one of the 15, we looked at we thought one was a fictious address, and we don’t know whether those email addresses were solely used or used by the family. Email is not a particularly fashionable method of communication anyway these….so I think, email will not have any sort of longevity as a bona fide method of communication.* (Staff 1).

[Email contact] *on the security, confidentiality, it doesn’t exist, obviously within* *NHS.net**and one or two other domains it’s secure, but once it leaves that, it isn’t secure at all. If I am asked to do so,* [send an email to a patient] *I always have that conversation with the patient around you understand that you are taking a risk I can’t protect your information once I press send. I’ve never had anyone who’s unhappy, I don’t think they understand the risk, all be it very small, of it being intercepted, but there is risk obviously it isn’t encrypted and it isn’t protected end to end.* (Staff 1).

#### Drivers and inhibitors

The drivers were seen as efficacy and efficiency. Improvements would reduce cost, improve communications, and reduce miscommunication and complaints.

*I think there are a number of key areas, so, one of them is about efficiency, so making communication more effective, i.e. it actually works, it gets to the right place, if it’s more acceptable to the patient then it is more likely to be effective, there is something about cost, we want to try to move to things that are cheaper like email and text, which are cheaper than old fashioned letters, things like traceability, so letters might go astray, I guess there is similar problems with email and text, but at least we’ve got different routes, so that efficiency and efficacy of communication and there’s also issues about new uses for communication and data use for things like research, dealing with patient concerns and professional concerns about how data is used and collected.* (Staff 2).

Drivers for the permissions section of the project were perceived as increased ability to identify patients interested in research, leading to increased recruitment and rapid uptake of research opportunities. Barriers to completing this type of work were identified as inertia, lack of interest, workload, and the fragmented nature of NHS digital systems.

Methods to incentivise PHC uptake of research, such as payment for recruitment to research, were discussed. Staff interviewees thought these could be further utilised; however, caution was expressed. Concern centred on patients being ‘turned off’ by practices being paid to find research participants that being contrary to the altruistic motive’s patient interviewees stated for taking part in research.

#### Outcomes and findings

Staff and patient interviewees suggested the relationship between the practice and the patients had affected the response rate in a positive way. Additionally, a large proportion of those who completed the survey online also completed the ‘Friends and Family’ questionnaire. This is an NHS wide survey carried out to aid the assessment of quality and available via the practice website. These were all completed with a favourable view of the practice. This reflected the patient interviewees who expressed a desire to give something back to the practice and feelings of autonomy and empowerment from being asked their views. Empowerment enables patient to become active participants in their healthcare, promoting understanding and informed choice. Increased empowerment leads to greater satisfaction and improved patient outcomes [16].

*That’s* [response rate] *very high*, *much higher than I was expecting…maybe 10* [%] *if we were lucky. I don’t know whether the idea of being able to express your preference for how you want to contacted is quite a draw for patients ‘cos that actually does make a difference for basically management and ease of their, even if they are not interested in research at all, I wonder if that was helpful. Or just that they like us.* (Staff 2).

There was an expectation that SMS would be the preferred contact method. However, reflecting the patient interviews, there was an understanding that there are those who will continue to require and prefer communication by letter. Both interviewees thought the project was feasible to carry out in a general practice as it had minimal impact on the wider practice team.

*Well I guess the people who want communication by post, communicating with them digitally isn’t going to be more effective anyway, I think as long as you don’t go down the route of saying if we had some sort of initiative, research initiative or direct health care initiative as long as you’re* [not] *saying we can only deliver that to people who have got a digital channel then I don’t think there is a major risk of alienating those people, as long as you are saying that now we have a health initiative let’s say we are going to target people for a flu vaccine, we are going to email and text, as long as you are making equal effort to contact the people who don’t have those digital channels by telephone or letter then I think that’s a reasonable approach.* (Staff 2).

## Discussion

The primary aim of this project was to increase the understanding of patient preferences, response rates and factors affecting these preferences and response rates. The aspiration was to use this knowledge to support more effective mechanisms of trial recruitment from primary care. The viability of creating a consent-for-contact registry was also assessed. A registry created with this method alone would contain 703 individuals which equals 15% of the eligible practice population. On its own, a registry of this size would not be viable as a method of trial recruitment. Permission to data share was most commonly given to NHS researchers, followed by university and commercial researchers. Combining the age groups shows a higher number of women giving permission to one or more organisations, although in the 70+ age group there were a higher number of males. Whilst this did not reach statistical significance it is worthy of reporting and of further exploration. Preferred contact method was closely correlated with age, with SMS messaging being the favoured overall mode, apart from the 70+ age group, who wished to be contacted by letter. Having an LTC did not appear to affect the permissions given.

Factors which may have affected the profile of the registry and therefore influence the transferability of the findings, include the geographical location, demographic and socioeconomic makeup of the setting. Typical of many areas in the North East of England, the practice area has suffered from the loss of heavy industry and long-term unemployment since the1980s. Relative deprivation was noted in 2015 to be worsening [17]. However, the practice population is stable, with only 250 new patient registrations in the last year, equalling 4.2% of the practice population. This enables lasting relationships to be built between primary health care professionals and patients. It was this relationship and the desire by patient participants to give something back that was thought to have a positive impact on the number of patients within the registry. The characteristics of local registries from areas with a different demographic and socioeconomic profile to the practice area may in fact differ and this merits further investigation. However, the population of the North East of England is estimated at 2.6 million [18] and if 15% of those agreed to join a regional registry 182,000 people, based in PHC, would be available for researchers to contact to offer involvement in research studies.

Additional measures that may aid recruitment from a PHC setting include focussing on studies led by NHS or universities and conducted in health promotion and on LTC. These were identified by patient interviewees as organisation that were trusted and research areas of interest. Approaches solely by commercial organisations are more likely to be mistrusted and perceived as overt commercialisation of the research process.

Whilst there is a push to move to email communication across the healthcare setting [19, 20] this work suggests it is the mode of approach least favoured by patients. The older demographic expressed a preference for contact by letter. Interview data suggests letters are associated with beliefs which increase the bona-fide nature of the content. Additionally, they are linked by the interviewees to subjects and ideas that require multiple reading, thinking and decisions. Email may be preferred by those who are already using it regularly, but there are concerns regarding the governance processes and the confidentiality of the material within the email [21]. We suggest initial approaches to individuals regarding recruitment to research studies should be made by letter and alternative contact mediums negotiated on an individual basis.

Work on a single dynamic platform which will allow contact and data-sharing preference settings to be recorded and updated as required is ongoing in the region [22]. Looking forwards, lessons can be learned from this initial small-scale project. Strategies to increase the number of patients responding need to be adopted to allow this approach to become impactful. As there was a degree of misinterpretation by patients regarding the focus of this project, we would suggest further work is needed on the materials used to communicate the use of registries to potential participants. For example, it may be worth splitting communications preferences from permission preferences to reduce the degree of misunderstanding and the initial volume of information given. This could possibly decrease the degree of confusion and conflation between information about data sharing, approaches to improve communication and ways to expand recruitment to research projects. Additional strategies which may increase the response rate would be the implementation a second contact. Further strategies include the removal of the commercial organisations option and replacing it with an option which refers to work in partnership with commercial organisations based within either the NHS or universities.

We suggest discussion with the research community regarding the demographic profile of the registries. It was thought that the older demographic profile was more likely to give permission due to the altruistic motives and feelings of reciprocity to the health care system [23, 24]. Those interviewed also spoke of cues to action [25] which prompted their interest in taking part in research. Further work needs to be undertaken to explore the motivations of differing demographic profiles and from that formulate strategies to engage them. In particular the younger profile who were markedly absent in this local registry. Furthermore, systems and processes need to build functionality that allows people to change their options in line with their personal circumstances. This would enable those who receive that ‘cue to action’ to opt into research which is relevant to them. It is possible that future work looking at a dynamic single medical record where patients can set their own data-sharing and contact preferences may prove more cost effective in setting up trusted research environments. Potentially, commercial companies who are currently working in this area could take this forward using App technology. It was previously thought the NHS App may take on this role but recent reports have suggested this may not be the current vision [26].

In the wider context, there is also an opportunity to link the registries to the NIHR INVOLVE [27] platform. Signposting would enable those motivated to seek out research opportunities for themselves. Approaches that increase the profile of research within PHC. should also be applied. Recruitment opportunities need to be better signposted, electronic ‘poster boards’ could be used to give information about current studies and practice websites utilised. A system could also be set up where by studies focussed on long-term conditions could communicate relevant studies to PHC staff (specialist nurses, district nurses, nurse practitioners, podiatrist, dieticians) who could then signpost patients towards any relevant projects. A system-wide approach is required to normalise the option of taking part in research as part of primary healthcare.

An additional and unexpected finding from this project suggests that current health communication trends could marginalise a subset of the population. The NNN response contains a significant number of women in the 70+ age group. When correlating this with the preferred contact method of letter in this group it suggests women are less confident with information technology and may mistrust data sharing activities. These could be called the digitally disenfranchised and appeared disempowered by the move to online services [28]. This finding is worth further exploration.

Whilst this has been a small-scale project, set in one locality in a single General Practice, it has directly influenced two further important developments. The first is a collaborative consent-for-contact registry across six federations in the North East, using text messaging rather than emails. The registry, called Research+Me (https://newcastlejro.com/take-part/researchplusme/), is managed by Newcastle Hospitals Trust and has been tremendously successful. In 3 months over 17,000 registrants have signed up and the registry has already been used to successfully recruit to trials. The second development resulted from the finding of this work that only a small proportion of patients would respond to a registry process; that the most inclusive method of screening patients would be to invite all potentially eligible patients as per the current PIC process. Keeping this PIC process, we have worked across a number of federations to create a ‘Super-PIC’ process which allows streamlined searches to be conducted across large populations by single point of contacts in each federation.

## Conclusions

The project was successful in setting up a primary health care-based registry of patients who had given researchers permission to contact them. The preferred contact method was SMS although letter was the contact method of choice for the over 70-year-old population. The survey methodology was not thought to be a viable process to build effective patient registries from a single practice, but have led to a region-wide collaboration to build a successful registry. The findings suggest the older demographic may be more likely to volunteer to take part. Studies conducted in health promotion and chronic disease may recruit better from PHC. Projects set within the NHS and academia were trusted. This methodology complements the work overtaken by local research alliances, and in particular the findings regarding targeted recruitment and preferred contact methods are directly transferrable to these alliances.

## Data Availability

The datasets used and analysed during the current study are available from the corresponding author on reasonable request.

## Abbreviations

SMS: Short Message Service
NHS: National Health Service
YYY: Permission to share with all 3 organisations
YYN: Permission to share with NHS and Universities
YNN: Permission to share with NHS only
YNY: Permission to share with NHS and Commercial
NYN: Permission to share with University only
NYY: Permission to share with University and Commercial
NNY: Permission to share with Commercial only
NNN: Declined permission to all 3 organisations
LTC: Long Term Condition
NIHR INVOLVE: National Institute for Health Research
